# Diagnosis, management and long term cardiovascular outcomes of phenotypic profiles in pulmonary hypertension associated with congenital diaphragmatic hernia

**DOI:** 10.3389/fped.2024.1356157

**Published:** 2024-03-25

**Authors:** Tejasvi Chaudhari, Nadia Schmidt Sotomayor, Rajesh Maheshwari

**Affiliations:** ^1^ Department of Neonatology, The Canberra Hospital, Canberra, ACT, Australia; ^2^ Australian National University Medical School, Australian National University, Canberra, ACT, Australia; ^3^ Department of Neonatology, Westmead Hospital, Sydney, NSW, Australia; ^4^ The Faculty of Medicine and Health, The University of Sydney, Sydney, NSW, Australia

**Keywords:** congenital diaphragmatic hernia, pulmonary hypertension, pulmonary hypoplasia, ventricular dysfunction, left ventricular hypoplasia, ductal shunt, atrial shunt, cardiac output

## Abstract

Congenital diaphragmatic hernia (CDH) is a developmental defect of the diaphragm resulting in herniation of viscera into the chest. This condition is characterized by pulmonary hypoplasia, pulmonary hypertension (PH) and cardiac ventricular dysfunction. PH is a key component of the pathophysiology of CDH in neonates and contributes to morbidity and mortality. Traditionally, PH associated with CDH (CDH-PH) is thought to be secondary to increased pulmonary arterial resistance and vasoreactivity resulting from pulmonary hypoplasia. Additionally, there is increasing recognition of associated left ventricular hypoplasia, dysfunction and elevated end diastolic pressure resulting in pulmonary venous hypertension in infants with CDH. Thus, hemodynamic management of these infants is complex and cautious use of pulmonary vasodilators such as inhaled nitric oxide (iNO) is warranted. We aim to provide an overview of different phenotypic profiles of CDH associated PH and potential management options based on current evidence and pathophysiology.

## 1 Introduction

Congenital diaphragmatic hernia (CDH) is a rare congenital anomaly secondary to a developmental defect of the diaphragm that produces the herniation of abdominal organs into the thorax of the fetus. This pathology affects approximately 1 in 3,500 liveborn infants (1). The anatomical defect of the diaphragm is more frequent on the left side (75%–90% of cases) (2). The herniation of abdominal contents into the chest during important stages of the embryonic and fetal development produces a complex condition characterized classically by pulmonary hypoplasia, pulmonary hypertension (PH), and more recently identified left ventricular dysfunction (3, 4). Despite considerable advances in antenatal and perinatal diagnosis and management, CDH-PH continues to be an important cause of neonatal morbidity and mortality and most challenging aspect of CDH care (3, 5). This article aims to describe different phenotypic profiles of CDH-PH and provide recommendations on diagnosis and management of these infants.

## 2 Definition of PH in CDH

Traditionally in children, PH is defined as a mean pulmonary arterial pressure (PAP) of ≥25 mmHg on cardiac catheterization (6, 7). Neonates have physiological PH during normal postnatal transition. Hence a simple pressure-based definition is not possible. Echocardiography or point of care ultrasound (POCUS) is the mainstay for diagnosis of CDH-PH as invasive testing is not practical or feasible. CDH-PH can be defined as raised PAP relative to systemic blood pressure resulting in dysfunctional pulmonary blood flow, poor oxygenation, cardiac dysfunction and/or systemic hypotension (3, 8).

## 3 Pathophysiology of PH and circulatory dysfunction in CDH

The pathophysiology of CDH-PH is multifactorial in nature contributed by abnormal pulmonary parenchymal and vascular development, pulmonary vasoreactivity and left ventricular hypoplasia and/or dysfunction as described below.

### 3.1 Pulmonary vascular remodeling

The anatomical abnormality of the diaphragm and subsequent herniation of abdominal contents during critical stages of lung development produces a mass effect that affects the growth of lung parenchyma, bronchial/bronchiolar branching, and the development of the pulmonary vasculature (3). On postmortem examination, ipsilateral lung has been found to be grossly hypoplastic (9). There is reduced bronchial and bronchiolar arborization, fewer alveoli, acinar hypoplasia, thick alveolar walls and increased interstitial tissue resulting in a reduced gas-exchange area (10). The abnormalities of pulmonary vasculature in CDH have been well described and include both structural and functional changes (3, 9, 10). Pulmonary arteries are decreased per unit lung volume. There is reduced pulmonary vascular branching accompanied by thickening of muscular media and adventitia (9–11). All these changes lead to a decreased anatomical vascular area for gas exchange. Multiple molecular pathways such as retinoic acid, nitric oxide, endothelin, vascular growth factors, micro RNAs, and impaired endothelial cell function have been implicated in the pulmonary vascular remodeling (3, 11). Functionally, the pulmonary vessels demonstrate abnormal vasoreactivity from an impaired endothelial response to vasoconstrictor and vasodilator signals and an imbalanced autonomic innervation (11, 12). Doppler studies of the fetal pulmonary flow across the pulmonary artery (PA) have shown increased pulsatility index consistent with increased pulmonary vascular resistance (PVR) (13). The severity of postnatal pulmonary hypertension is difficult to predict antenatally despite many efforts to review prenatal anatomy and relationship between the size of the defect and the size of the fetal lungs and heart (14). These structural and functional changes in pulmonary vessels prevent vasodilation and normal transition to postnatal circulation after birth resulting in increased pulmonary arterial pressure or pre-capillary pulmonary hypertension (15).

### 3.2 Right ventricular (RV) dysfunction

Increased afterload from persistent or severe PH in infants with CDH results in RV hypertrophy and dilatation. RV hypertrophy reduces compliance and diastolic filling whilst increasing oxygen demand. Increased RV pressure can also reduce coronary perfusion and lead to myocardial ischemia. Furthermore, a decreased preload of the right ventricle is frequent in ventilated infants after birth due to elevated mean airway pressure and poor venous return. RV dilatation can lead to shifting of interventricular septum towards the left ventricle impacting left ventricular (LV) performance. The net result is RV or biventricular dysfunction and further reduction in pulmonary and systemic blood flow (8, 16). Both systolic and diastolic dysfunction have been observed in studies on echocardiography early after birth and after surgery and have been correlated with need for extracorporeal membrane oxygenation (ECMO) and mortality (16–18).

### 3.3 Left ventricular (LV) hypoplasia and dysfunction

There is increasing recognition of LV dysfunction in infants with CDH due to severe pulmonary arterial hypertension and/or fetal LV hypoplasia (8).

Right heart dysfunction directly affects the left ventricle due to the interdependence of both structures that share anatomical elements such as muscular fibers, interventricular septum (IVS), and pericardium. Shifting of the IVS to the left directly impacts the filling of the LV affecting the cardiac output of the left heart (19–21).

Not all LV dysfunction in CDH is due to RV dysfunction. Recent studies have shown isolated or primary LV dysfunction without RV dysfunction (22, 23). Several factors may contribute to primary LV dysfunction as given below:

• *Fetal LV hypoplasia.* The congenital defect of the diaphragm with resulting visceral herniation during essential stages of development produces a mass effect in the chest that also causes variable degrees of hypoplasia of the heart, especially of the left ventricle. This effect seems to be independent of the size of the diaphragmatic defect (3, 4). Moreover, the decreased flow of blood through the PA during fetal life with subsequent reduced blood flow to the left heart affects the normal development of the left heart considerably (24). Another described factor is the redirection of blood flow from the ductus venosus and inferior vena cava due to the mediastinal shift. Normally, this flow is preferentially directed to the patent foramen ovale (PFO) and therefore acts as significant preload for the left ventricle. In infants with CDH, the redirection results in this blood streaming away from the PFO and going towards the right ventricle. A large portion of this blood subsequently goes through the patent ductus arteriosus (PDA) into the descending aorta. Both these factors (reduced pulmonary blood flow and streaming of blood away from PFO) are hypothesized to cause less growth of the left ventricle (25). Furthermore, it seems that infants with CDH have an abnormal embryonic and fetal development of the heart. The structural anatomy of the left heart of an infant with CDH includes a narrowing and elongated left ventricle (4).
• *Acute changes in LV loading.* Primary LV dysfunction is also related to acute changes in loading conditions after birth. The left ventricle is suddenly faced with a higher afterload with the removal of the low-resistance placenta. If the LV preload remains low due to reduced pulmonary blood flow, this combination of reduced preload and increased afterload, exacerbates LV dysfunction. The LV dysfunction is also self-perpetuating as the resultant hypotension leads to potential reduction in coronary blood flow (4).
• *Acidosis and hypoxemia*, as a result of severe PH, interatrial and ductal shunting or hypotension can cause LV dysfunction.

LV dysfunction usually presents immediately after birth and can cause decreased systemic blood flow and hypotension. In addition, LV dysfunction can increase left atrial and pulmonary venous pressure due to reduced LV end diastolic volume. This in turn will lead to pulmonary venous hypertension or post-capillary pulmonary hypertension.

## 4 Phenotypic profiles of PH in CDH

Phenotypic profiling of CDH-PH is based on contributing pathophysiological factors that are important to consider to guide physiology-based management and prognostication. In addition to clinical assessment, an echocardiographic evaluation is critical for phenotypic profiling of CDH-PH (1). A POCUS or echocardiography should be performed in all infants with CDH after initial stabilization. The scan should include structural assessment for congenital heart disease, assessment of PH, PAP measurements, ventricular size and function and shunts across the PDA and PFO.

**Figure 1:**
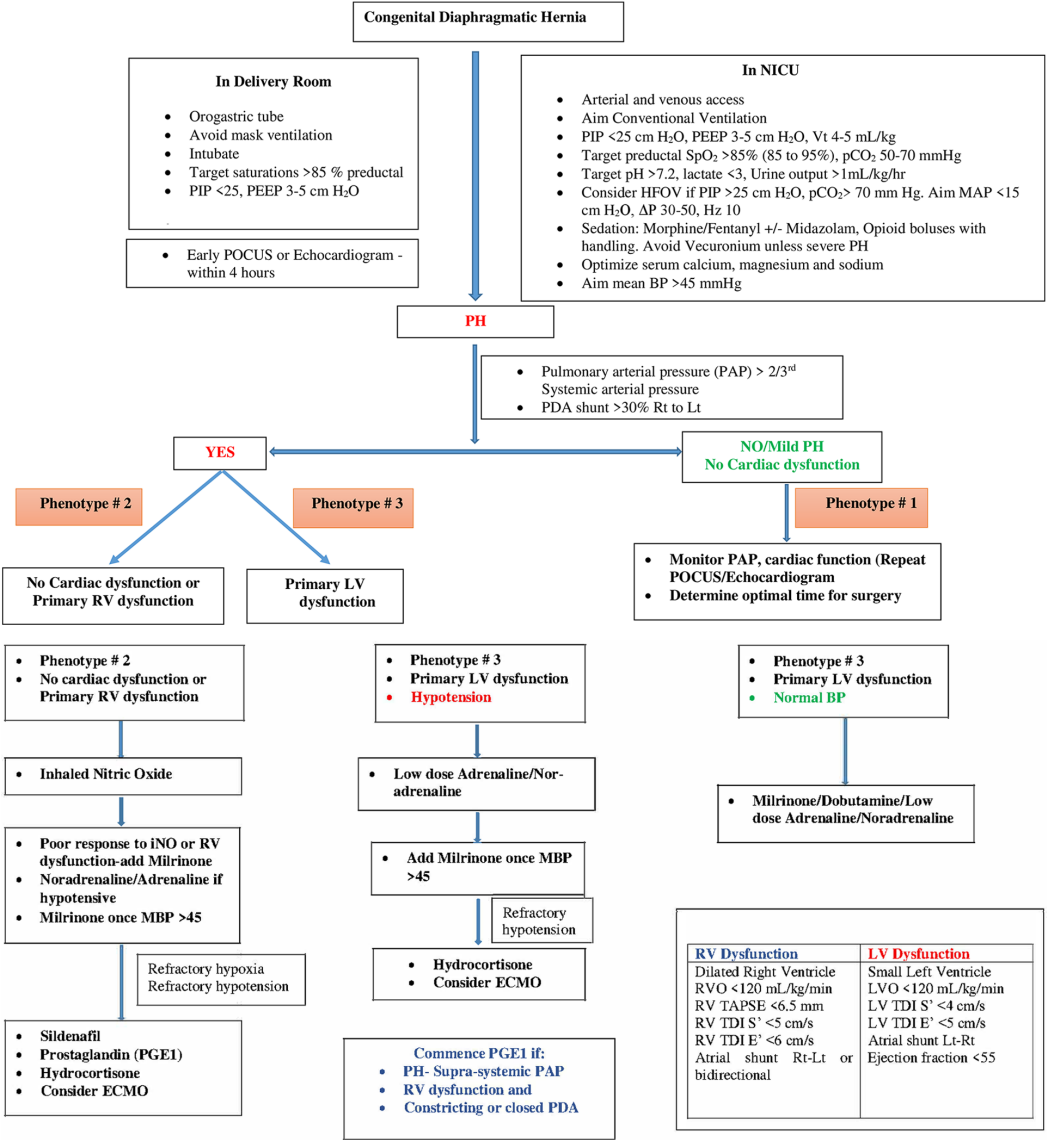
Flowchart for management of CDH and different phenotypes of CDH-PH.

Table 1 summarizes various echocardiographic measurements described in literature in infants with CDH (26–28). Each measurement has some limitations and there is no single gold standard measure (4). In our practice we use a combination of these measures to determine predominant phenotype and guide a physiology-based management (Figure 1).

**Table 1:** Echocardiographic measurements to assess pulmonary hypertension and cardiac function in CDH.

Parameter	Measurement	Notes
Pulmonary artery pressure		
Peak tricuspid regurgitation velocity (TRmax)	- Estimates RV peak systolic pressure from TR Jet velocity using modified Bernoulli equation - RV systolic pressure = 4 (TRmax)^2^ + Right atrial pressure Right atrial pressure is usually estimated at 2–5 mm Hg	- Not reliable in absence of right ventricular failure or right ventricular outflow tract obstruction - TR jet may not always be observed or well demarcated
Patent ductus arteriosus (PDA) flow	- Doppler assessment of PDA flow and velocity - Estimation of systolic pulmonary arterial pressure when right to left shunt lasts ≥ 30% cardiac cycle: Systolic PAP = 4 (VmaxDA)^2 ^+ Systolic systemic arterial pressure	Ductal shunt is right to left or bidirectional in PH
Interventricular septum shape and position	Measured from parasternal short axis view at mid LV level	- Interpretation based on shape O shaped left ventricle- RVP <50% LVP D shaped left ventricle- RVP 50–100% of LVP Crescent shaped left ventricle- RVP ≥ 100% LVP
Pulmonary artery acceleration time: right ventricular ejection time (PAAT:ET)	Measured from doppler waveform of main pulmonary artery during systole	- Validated, feasible and reproducible measure - Timing and duration change with increasing PA pressure PAAT <90ms- pulmonary vascular disease PAAT <40 msec- severe pulmonary hypertension Normal PAAT:RVET ratio - > 0.3 PAAT:RVET <0.23 is indicative of increased PAP
Right ventricular Function		
Tricuspid annular plane systolic excursion (TAPSE)	Obtained from the 4-chamber view using the M mode with the cursor aligned along the direction of the lateral annulus	- Measure of RV longitudinal function - Normal value in neonates 1,500–2,500 g is 0.45 ± 0.03 cm, & in neonates 2,500–3,600 g is 0.8 ± 0.16 cm - TAPSE < 4 mm is predictive of need for ECMO and death in neonates with PPHN
Tissue doppler imaging (TDI) of diastolic and systolic myocardial velocities	Measures longitudinal systolic (s’) and diastolic velocities (e’ and a’) in basal myocardium of RV near lateral tricuspid valve leaflet	- Assesses both systolic and diastolic RV function - Angle dependent - Relatively load independent
Right ventricular output (RVO)	- Measured in parasternal long axis- right heart view at the level of the pulmonary valve - Calculated from cross-sectional area of pulmonary valve and VTI using doppler of main pulmonary artery just distal to the pulmonary valve	- Affected by intra and extra cardiac shunts - High observer variability - RVO <120 ml/kg/min is considered low or abnormal
Myocardial performance index (MPI)	Global measure derived from pulse doppler or tissue doppler imaging	- Also referred to as Tei index. - Highly load dependent - Does not differentiate systolic from diastolic dysfunction - RV dysfunction will increase time of isovolumic phases and therefore lead to higher MPI
Strain assessment using speckle tracking echocardiography (STE)	Quantitative assessment of global & regional deformation (strain, strain rate, twist) in multiples planes	- Need specific hardware and software and experienced operators - Not routinely available
Left ventricular function		
Left ventricular ejection fraction (LVEF)	- Percentage of change in LV volume from end- diastole to end-systole - Calculated using M-mode in parasternal long axis or short axis views by measuring left ventricular end diastolic and systolic diameters	- Angle- and load-dependent, inter-observer variability, affected by septal shape and dysfunction. EF >55%—normal EF 41%–55%—slightly reduced EF 31%–40%- moderately reduced, & EF < 30% markedly reduced
TDI myocardial velocities	Measures longitudinal systolic (s’) and diastolic velocities (e’ and a’) in basal myocardium of LV near lateral mitral valve leaflet	- Assesses both systolic and diastolic LV function - Angle dependent - Relatively load independent
Left ventricular output (LVO)	Calculated from cross sectional area of aortic valve (parasternal long axis view) and VTI from doppler in ascending aorta just distal to the aortic valve (apical five-chamber view)	- Affected by intra and extra cardiac shunts - High observer variability - LVO <120 ml/kg/min is considered low or abnormal
Myocardial performance index (MPI)	Global measure derived from pulse doppler or tissue doppler imaging	- Highly load dependent - Does not differentiate systolic from diastolic dysfunction
Strain assessment using speckle tracking echocardiography (STE)	Quantitative assessment of global & regional deformation (strain, strain rate, twist) in multiples planes	- Need specific hardware and software and experienced operators - Not routinely available
Shunt physiology		
PDA and interatrial shunting	Doppler assessment of direction & velocity of blood flow	- A bidirectional shunt through PDA with a systolic right-to-left duration ≥30% of the total cardiac cycle is considered non-physiologic and likely to represent PPHN - Atrial shunting depends on relative RV to LV compliance

Based on the assessment of PH including atrial and ductal shunting, and cardiac function, three CDH-PH phenotypes have been described (Table 2).

**Table 2:** Phenotypic profiles of CDH-PH.

Variable	Phenotype 1	Phenotype 2	Phenotype 3
PH	Mild or none	Present, primarily related to pulmonary arteriolar changes	Present, primarily related to pulmonary venous hypertension
Cardiac function	Normal	None/RV dysfunction with or without secondary LV dysfunction	Primarily LV dysfunction, may have biventricular dysfunction
Hypoxemia	Related to pulmonary causes e.g. VQ mismatch if present	Pulmonary causes, right to left atrial and ductal shunting. Significant pre- and post-ductal SpO_2_ difference of >10% (pre > post)	Pulmonary causes (including pulmonary congestion), right to left ductal shunting. Significant pre- and post-ductal SpO_2_ difference of >10% (pre > post)
Echocardiography findings	Normal biventricular function, left to right ductal and atrial shunting	1. Changes of PH (TR jet, right to left ductal and atrial shunting, IVS flattening or bulging into LV) 2. May have changes of RV dysfunction (reduced TAPSE and RVO, changes on TDI- see Figure 1) 3. May show secondary LV dysfunction (reduced EF, reduced LV output, changes on TDI- see Figure 1)	May show many of the same changes as in phenotype 2 but with left to right atrial shunting, smaller LV
Therapy	Achieve adequate lung inflation	1. Normal cardiac function: iNO 2. iNO nonresponse- milrinone, sildenafil 3. RV dysfunction: iNO, milrinone, low dose adrenaline, PGE1 4. Hypotension: adrenaline/noradrenaline followed by milrinone. Consider hydrocortisone	1. Avoid inhaled NO 2. LV dysfunction: milrinone, dobutamine, low dose adrenaline 3. Hypotension: adrenaline/noradrenaline followed by milrinone. Consider hydrocortisone
ECMO		As rescue therapy	As rescue therapy

### 4.1 Phenotype 1

#### 4.1.1 Mild or no PH with normal cardiac function

In this phenotype, there is no clinically significant difference between pre-ductal and post-ductal oxygen saturation. If there is hypoxia, this is related to pulmonary causes such as atelectasis causing ventilation-perfusion (VQ) mismatch, and usually there is clinical response to appropriate changes in ventilatory settings.

On echocardiography, the shunting across the PDA and atria is predominantly left to right. There is either no evidence of PH, or features of only mild PH, and biventricular function is normal.

### 4.2 Phenotype 2

#### 4.2.1 Pulmonary arterial hypertension (pre-capillary PH) with either no cardiac dysfunction, or primary RV dysfunction with or without secondary LV dysfunction

The PH in this phenotype is primarily related to the changes in the pulmonary arterioles. In this situation, there is likely to be significant difference (>10%) between pre- and post-ductal oxygen saturation (pre > post).

Echocardiography shows features of elevated pressure in the PA, such as tricuspid regurgitation (TR) jet with increased velocity in Doppler, dilated right ventricle, and septal flattening or bulging into the left ventricle. RV function may be preserved or there may be systolic and/or diastolic RV dysfunction. Secondary LV dysfunction may be seen as well as there is ventricular interdependence between the right and left ventricle. In addition, if the IVS bulges into the left ventricle due to elevated RV pressures, this may lead to LV diastolic dysfunction and impaired filling.

Depending on the level of pulmonary arterial pressure compared to the systemic pressure, the shunting through the PDA may be low velocity left to right, bidirectional, or pure right to left. In the setting of RV dysfunction and preserved LV function, shunting at atrial level is right to left and this shunting contributes to pre-ductal hypoxemia.

### 4.3 Phenotype 3

#### 4.3.1 Pulmonary venous hypertension (post-capillary PH) with primary LV dysfunction

The post-capillary PH is primarily related to the underdevelopment of the left ventricle which affects left CDH more commonly than right CDH. The net effect of the LV dysfunction is an elevation in the left atrial pressures which leads to left to right flow at the PFO level. The ductal shunting could be bidirectional, or right to left due to increased pulmonary arterial pressure in relation to the systemic arterial pressure. This combination of bidirectional or right to left ductal shunting, and left to right PFO shunting, is important to recognize as pulmonary vasodilators such as inhaled nitric oxide (iNO) are unlikely to be helpful and may even cause harm by increasing the risk of pulmonary edema and hemorrhage. This is the most likely explanation for the lack of benefit shown for iNO in the treatment of CDH in a randomized controlled trial (RCT) published in 1997 (29). This is further supported by a more recent retrospective study (30) which concluded that treatment with iNO was associated with improved oxygenation and reduced need for extracorporeal membrane oxygenation (ECMO) only in a subpopulation of infants with CDH who have normal LV systolic function.

A publication from the CDH Study Group (23) that collected cardiac function prospectively (within 24–48 h of age) in 1,173 infants with CDH showed normal cardiac function in 61% of the cases, RV dysfunction in 15%, LV dysfunction in 5% and biventricular dysfunction in 19% of the cases. Supra-systemic PH was documented in 32% of the cases, but its frequency was more common (57%) when there was biventricular dysfunction. Survival varied according to the cardiac dysfunction with survival figures of 80%, 74%, 57% and 51% in the categories of normal cardiac function, RV dysfunction, LV dysfunction and biventricular dysfunction respectively.

Putnam et al. (31), investigated spectrum of iNO use in infants with CDH utilizing CDH Study Group data collected between January 2007 and December 2014. Their study showed that iNO use was very common (60.8%) and 36% of infants without PH received iNO. Nitric oxide use was significantly associated with increased mortality.

The above evidence suggests that early echocardiogram is essential to identify PH and its appropriate phenotype and that a targeted pathophysiology-based management may be beneficial and improve outcomes. We recommend performing an echocardiogram within the first 4 h after birth- ideally immediately after stabilization. Subsequent echocardiograms should be done every 6–12 h during the transitional period (24–48 h) or following clinical changes or treatment to determine changing physiology and assess response to treatment.

## 5 Therapeutic strategies to manage phenotypic profiles of PH in CDH

Given its multifactorial nature, management of CDH and CDH-PH is complex and depends on predominant pathophysiologic or phenotypic profile. Key principles of management applicable to all 3 phenotypes include maintaining oxygenation, blood pressure and cardiac output; optimizing lung recruitment (avoiding overdistension); and early diagnosis and management of PH and/or associated cardiac dysfunction.

### 5.1 General measures

Current observational evidence suggests better outcomes for infants with CDH managed medically and deferring surgical repair until their respiratory status and PH have improved (32, 33). For prenatally diagnosed CDH, a nasogastric tube should be placed immediately after delivery to decompress the stomach and intestines, and the infant should be intubated in the delivery room without mask ventilation. Avoidance of ventilator-induced lung injury with gentle ventilation strategies is the cornerstone of respiratory management in infants with CDH (Figure 1). A multicenter randomized trial (VICI trial) compared conventional mechanical ventilation to high-frequency oscillatory ventilation (HFOV) as the initial mode in infants with CDH. The study showed shorter ventilation time and lesser need for ECMO in the conventional ventilation group compared to HFOV group (34).

The approach to sedation is generally similar to the management of infants with non-CDH related PH. Sedation should be titrated to maintain spontaneous breathing if possible, and muscle relaxation should only be used when there is infant-ventilator dyssynchrony despite optimal sedation and adequate ventilatory settings (35).

### 5.2 Phenotype 1

#### 5.2.1 Mild or no PH with normal cardiac function

As there is no or mild pulmonary hypertension, hypoxia is generally due to parenchymal lung disease or atelectasis and is managed with adequate lung recruitment in addition to general measures described above.

### 5.3 Phenotype 2

#### 5.3.1 Pulmonary arterial hypertension (pre-capillary PH) with either no cardiac dysfunction, or primary RV dysfunction with or without secondary LV dysfunction

Management in this situation targets reduction in PVR with adequate sedation and lung recruitment (avoiding overdistension and derecruitment), and the use of pulmonary vasodilator therapies such as iNO, sildenafil and/or milrinone (4, 8). Use of prostaglandin E1 (PGE1) may also be considered if there is evidence of RV dysfunction as this reduces RV afterload, though this can result in post-ductal hypoxemia.

Inhaled nitric oxide is the most common pulmonary vasodilator used for CDH-PH (6). However, evidence from both RCT and CDH databases has failed to show an improvement in survival or decrease need for ECMO (29, 36). One possible explanation is the associated LV dysfunction observed in these infants. Pulmonary vasodilation from iNO could lead to LV failure and pulmonary edema by increasing the venous return (30). Other possible reasons for lack of iNO benefit could be the abnormal development of the pulmonary vasculature, blunted nitric oxide signaling pathway, or overexpression of vasoconstrictors such as endothelin 1 (37) in infants with CDH. Sildenafil, a phospho-diesterase 5 inhibitor, acts on the same pathway to prevent breakdown of cyclic GMP and augment action of nitric oxide. A recent international multicenter RCT (CoDiNOS trial) is currently comparing intravenous sildenafil and iNO for treatment of PH in infants with CDH (38).

Milrinone, a phosphodiesterase (PDE3) inhibitor, is another vasodilator that acts by targeting the cyclic AMP pathway. In addition, it has direct positive inotropic and lusitropic actions on the heart, which may benefit infants with coexisting RV dysfunction (39). Studies on use of milrinone in infants with CDH have shown improvement in oxygenation and cardiac function especially in combination with iNO (40, 41). A multicentre pilot RCT on impact of milrinone on oxygenation index in CDH is currently underway (42). It is important to consider that milrinone can cause systemic vasodilation and hypotension.

Systemic vasoconstrictors may be needed to support systemic vascular resistance (Figure 1). Noradrenaline has been demonstrated to reduce pulmonary/systemic pressure ratio in non-CDH neonatal PH and is our preferred inotrope to improve blood pressure in this phenotype (43).

Prostaglandin E1 (PGE1) is a potent pulmonary vasodilator and is also used to maintain ductal patency. In infants with severe PH, the ductus acts as a “pop-up” valve for a strained or failing right ventricle (44). In our practice we use PGE1 in infants with supra-systemic pulmonary hypertension and RV dysfunction with a constricting or closed duct.

We recommend commencing iNO as first line treatment for this phenotype. If there is no response within 30–60 min or if there is evidence of RV dysfunction, we recommend adding milrinone. If the baby is hypotensive, we recommend commencing low dose adrenaline or noradrenaline to improve mean blood pressure (MBP) > 45 mm Hg prior to adding milrinone. Sildenafil should be considered in patients with refractory pulmonary hypertension (i.e., unresponsive to inhaled nitric oxide) or as an adjunct when weaning inhaled nitric oxide.

### 5.4 Phenotype 3

#### 5.4.1 Pulmonary venous hypertension (post-capillary PH) with primary LV dysfunction

With LV dysfunction, there is increase in the left ventricular end diastolic pressure resulting in increased left atrial pressure and pulmonary venous hypertension. In this phenotype, pulmonary vasodilator therapies could lead to clinical deterioration by increasing blood flow to a failing left ventricle (30). Therefore, the treatment of PH in this phenotype is directed towards improving LV function and avoiding excessive LV afterload using inotropes such as dobutamine and low dose adrenaline (4, 8). Fluid boluses should be avoided unless there is evidence of hypovolemia.

Milrinone may also improve LV function by a combination of factors such as reducing LV afterload and improving LV contractility and diastolic relaxation (39). Infants with LV dysfunction are often hypotensive. In our practice, we commence low dose adrenaline (0.05–0.1 microg/kg/min) or noradrenaline (0.05–1 microg/kg/min)to improve blood pressure prior to commencing milrinone (Figure 1). In addition to a synergistic inotropic effect, these systemic vasoconstrictors may improve coronary blood flow (8). Use of PGE1 may also assist with improving systemic flow to the lower half of the body albeit at the cost of reduced oxygenation (8, 44). It is important to closely monitor cardiac performance through serial ultrasound scans in infants on multiple inotropes.

Hydrocortisone is often used as an adjunct treatment for hypotension in infants with CDH. Cortisol insufficiency is very common in these infants and it is seen in up to 2/3rd of cases. Cortisol supplementation can improve both cardiac output and systemic vascular resistance (45).

ECMO use in infants with CDH should be considered in cases with severe PH or respiratory failure and severe cardiac dysfunction unresponsive to maximal medical treatment. LV dysfunction typically is a transient phenomenon and resolves in the first few days of life (46). ECMO can provide time for the heart function to recover. Infants with severe pulmonary hypoplasia and irreversible pulmonary vasculature abnormalities may be refractory to weaning from ECMO.

Infants with biventricular dysfunction are the most challenging to manage and have the least survival rate (23). Our approach is to commence a trial of treatment based on the worst affected ventricle with close hemodynamic monitoring. Treatment is then guided by appropriate clinical response. These babies should be referred for ECMO earlier.

## 6 Long term cardiovascular outcome in CDH-PH

PH is one of the most important prognostic factors in CDH and the prognosis is worse with greater severity of PH. In a prospective multicenter CDH birth cohort (47), echocardiography was performed at 1 and 3 months of age. PH was classified into mild/none (RV systolic pressure less than half systemic), moderate (RV systolic pressure half to two thirds systemic) and severe (RV systolic pressure more than two thirds systemic). In the absence of TR jet, ventricular septal position and movement as well as RV function and size, were used to estimate PH severity. The mortality at discharge with mild, moderate, and severe PH at 1 month of age was 1.4%. 7.4% and 56.1% respectively. Whilst PH is responsible for a substantial proportion of early mortality due to CDH, it is also linked to late mortality (death after 1 year of age). In a Swedish study encompassing 25 years (48), 49 of 251 (19.5%) CDH patients died. Of these deaths, 7 (14.3%) occurred after 1 year of age. Two of these 7 deaths were attributed to PH with the oldest PH-associated death observed in a 9-year-old child.

In a follow-up study during infancy (49), PH was assessed using echocardiography and ECG at 6 and 12 months of age. In this study cohort of 52 infants, whilst 71% were diagnosed with PH at hospital admission, only 8% of CDH infants had persistence of PH during later infancy. Those with persistent PH had a higher frequency of using supplemental oxygen, tube feeding and medications (diuretics, sildenafil, bosentan) at the age of 6 months, though by the age of 12 months, these differences were in general not significant (except for the use of sildenafil which remained higher in the persistent PH group). In another echocardiography-based study (50), PH was assessed at 1 and 3 months post-repair and then during follow-up with a median age of 26 months. PH was defined as RV systolic pressure >0.5 systemic systolic pressure. The prevalence of PH at 1 month, 3 months and at longer term follow-up was 38.6%, 25.6% and 16% respectively. These differences in PH prevalence may be due to differences in CDH severity and the criteria used in diagnosing PH.

In a recent systematic review (51), authors have investigated the prevalence of adverse cardiopulmonary outcomes in CDH survivors at >2 years of age. These outcomes included pulmonary function testing, indices of PH and RV function, risk of chronic obstructive pulmonary disease, functional outcomes (exercise tolerance, health related quality of life) and radiological outcomes. They found thirteen eligible studies which investigated PH in CDH survivors. Of these, six studies reported on prevalence of PH in CDH survivors between 2 and 5 years of age. Echocardiography was used in all these studies. Rates of PH were noted to be ranging from 4.5% to 38%. Importantly, none of the studies which included participants with an average age of >5 years reported any PH. The authors discussed that this reduction in PH after the age of 5 years may be due to true reduction in frequency of PH with increasing age; however, some of the reduction could also be due to late PH-associated deaths.

Follow-up studies have also looked at RV function in CDH survivors during early childhood. In a case-control study (52), authors investigated RV function at a mean age of about 6 years with strain analysis and tissue Doppler imaging (TDI). Impairment of RV systolic and diastolic function was noted in CDH survivors with significant reduction in TDI velocities. Schwartz et al. (53) evaluated 21 CDH survivors (all received ECMO in the newborn period) at a mean age of 3.2 years. Eight of 21 (38%) patients met echocardiographic criteria for PH. Six of 21 (29%) had either right axis deviation or right ventricular hypertrophy on ECG; two of these also had PH on echocardiography. In a similar follow-up study of “CDH treated with ECMO” survivors (54), 18 children were seen at 1 and 2 years of age. ECG showed features of RV hypertrophy in a third of these children.

In a case-control study, the authors compared MRI based cardiac function and pulmonary arterial flows in 12 babies with CDH with normal age and body size matched controls at a median age of 12 years. Interestingly, while the ECG and echocardiography in CDH cases was normal, there was evidence of ventricular dysfunction and reduction in stroke volume with compensatory increase in heart rate. Flow, acceleration time and cross-sectional area of the left pulmonary artery were reduced. Long-term significance of these changes remains unclear however (55).

Some studies have reported on medication use for PH during longer-term follow-up. In a cohort of 17 surviving patients (severely affected with predicted lung volume <15%) (56), two children had PH at the age of 3 years and were receiving supplemental oxygen and sildenafil therapy. In a Canadian follow-up study (57), 2 of 44 (4.5%) surviving patients had PH and were receiving IV pulmonary vasodilator therapy by 2 years of age. Similarly, in the follow-up study by Garcia et al. (50), all the infants with PH remained on long-term therapy with either sildenafil or inhaled/IV prostanoids.

In summary, PH is linked to a substantial proportion of mortality in CDH patients. The prevalence of PH in CDH survivors decreases over time and there are no reports describing significant PH in CDH survivors at school age or beyond.

## 7 Summary

In conclusion, cardiac dysfunction is an important component of CDH-PH pathophysiology. Pre- and post-capillary mechanisms of increased pulmonary vascular resistance contribute to variable clinical phenotypes of right and left ventricular dysfunction. Early and regular assessment of pulmonary pressures and cardiac function is important to understand the underlying predominant phenotype and guide targeted, individualized treatment and may lead to improved outcomes in this challenging condition.
